# Acidity‐Mediated Metal Oxide Heterointerfaces: Roles of Substrates and Surface Modification

**DOI:** 10.1002/adma.202512804

**Published:** 2025-10-04

**Authors:** Gyu Rac Lee, Thomas Defferriere, Jinwook Kim, Han Gil Seo, Yeon Sik Jung, Harry L. Tuller

**Affiliations:** ^1^ Department of Materials Science and Engineering Massachusetts Institute of Technology Cambridge MA 02139 USA; ^2^ Department of Materials Science and Engineering Northwestern University Evanston IL 60208 USA; ^3^ Department of Materials Science and Engineering Dankook University 119 Dandae‐ro, Dongnam‐gu Cheonan‐si Chungnam 31116 Republic of Korea; ^4^ Department of Materials Science and Engineering Korea Advanced Institute of Science and Technology 291 Daehak‐ro Yuseong‐gu Daejeon 34141 Republic of Korea

**Keywords:** electrical conductivities, heterointerfaces, nanostructured functional oxides, praseodymium‐doped ceria nanowire arrays, space charge potentials, surface acidity

## Abstract

Although strong modulation of interfacial electron concentrations by the relative acidity of surface additives is suggested, direct observation of corresponding changes in surface conductivity, crucial for understanding the role of local space charge, is lacking. Here, a model platform comprising well‐aligned mixed ionic‐electronic conducting Pr_0.2_Ce_0.8_O_2‐δ_ nanowire arrays (PCO_NA_) is introduced to show that acidity‐modulated heterointerfaces predict electron depletion or accumulation, resulting in tunable electrical properties. Three orders of magnitude increased PCO_NA_ conductivity are confirmed with basic Li_2_O infiltration. Moreover, the relative acidity of the insulating substrate supporting the PCO_NA_ strongly influences its electronic properties as well. This strategy is further validated in purely ionic‐conducting nanostructured ceria as well as PCO_NA_. It is suggested that observed conductivity changes stem not only from acidity‐mediated space charge potentials at heterointerfaces but also from grain boundaries, chemically‐modulated by cation in‐diffusion. These findings have broad implications for how substrate and surface treatment choices can alter the conductive properties of nanostructured functional oxides.

## Graphical abstract

The role of local space charge, not only at heterointerfaces, but also at grain boundaries, influenced by surface acidity modulation is elucidated through model platforms composed of Pr‐doped ceria (mixed ionic‐electronic conducting oxide) nanowire arrays. The large potential impact of surface acidity on the electronic properties of nanostructured functional oxides is illustrated.

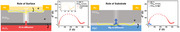

## 1 Introduction

Heterointerfaces have long been recognized as having a profound influence on the electronic properties of semiconducting materials, and their prudent engineering has been vital for the development of a broad variety of microelectronic devices including diodes, transistors, and solar cells.^[^ ^1^, ^2^, ^3^ ^]^ This stems from the ability of heterointerfaces to induce band bending, modify charge carrier distributions, and create built‐in electric fields that govern the electronic behavior at interfaces.^[^ ^4^, ^5^ ^]^ The same can be said for ionic materials, where heterointerfaces can lead to extraordinary properties arising from space charge effects with resultant defect redistribution. For example, the formation of 2D electron gases at the heterointerfaces of insulating oxides and the emergence of superconductivity of relevance for quantum applications have been, in part, associated with the accumulation of oxygen vacancies acting as electron donors.^[^ ^6^, ^7^, ^8^, ^9^ ^]^ In more conventional systems, these characteristics can be highly beneficial for enhancing the performance of energy storage and chemical conversion applications, including electrocatalysis, fuel cells, and batteries.^[^ ^10^, ^11^, ^12^ ^]^

One representative work by Sata et al. demonstrated marked increases in ionic conductivity with increases in interface density of CaF_2_ and BaF_2_ heterostructures.^[^ ^13^ ^]^ By reducing the heterointerface spacing to below 50 nm, they observed the predicted mesoscopic size effect and revealed that the overlap of space charge regions at nanoscale interfaces can lead to anomalous transport behavior. These findings highlight that precise engineering of the heterointerface enables modulation of the flow of ionic as well as electronic charges. However, studies that successfully exemplify the role of heterointerface space charge in controlling electronic or ionic transport in metal oxides remain limited, except for a few restricted specific compositions.^[^ ^14^ ^]^ This is due to difficulties in identifying material combinations with appropriate space charge characteristics and the inherent challenge of distinguishing their role from coexisting factors, such as strain,^[^ ^15^, ^16^ ^]^ atomic reconstruction,^[^ ^17^ ^]^ or adsorbate‐induced charge localization^[^ ^18^, ^19^, ^20^ ^]^ at heterointerfaces. Recently, Steinbach et al. developed a physicochemical model to predict space charge properties at heterointerfaces between various oxides and undoped SrTiO_3_ single crystals.^[^ ^21^ ^]^ They were able to relate the experimentally measured space charge potential to defect thermodynamics and the reducibility of oxides. While offering a widely applicable framework, the model depends highly on detailed knowledge of defect formation energies and the reducibility of oxides, not always readily available. Moreover, their analysis employs mixed ionic and electronic conducting (MIEC) materials requiring gas‐phase equilibrium, necessitating fast kinetics, thereby limiting applicability to high temperatures. Developing simpler material selection criteria for predicting the space charge properties of oxide‐based heterointerfaces would go a long way toward providing a practical framework for engineering such composite systems.

A previous study on surface‐infiltrated heterostructures by the authors demonstrated that the acidity of binary oxides acts as a sensitive descriptor for predicting the surface oxygen exchange rate (*k*_chem_) in MIEC electrodes.^[^ ^22^ ^]^ This was attributed to near‐surface electron depletion or accumulation, driven by the modulated space charge potential at the heterointerface, which correlated the relative Smith acidity of the surface infiltrated binary oxides relative to that of the host MIEC electrode.^[^ ^22^, ^23^, ^24^, ^25^ ^]^ Building on this insight, the Smith acidity scale, or equivalently the relative work functions of the oxides, serves as a powerful descriptor of electronic and ionic properties induced by local space charges formed at a broader category of heterointerfaces. In a broader sense, it also encompasses the same fundamental enthalpy relation described by Steinbach et al.^[^ ^21^ ^]^ This serves to provide a rational and useful guideline for materials selection that elucidates the relation between local space charge and conductivity. We furthermore note that the Smith acidity is derived in terms of an oxide's tendency to accept an oxygen anion from a basic oxide during a chemical reaction and calculated using enthalpy changes during such reactions. This framework is consistent with the framework derived by Steinbach et al., as mentioned above.

Although changes in the electrical conductivity of porous Pr_0.1_CeO_0.9_O_2‐δ_ layers upon acidic/basic binary oxide infiltration were also assessed, they were modest (10–25%),^[^ ^22^ ^]^ leading several studies to question the space charge model, despite similar trends being observed in electrode polarization with controlled surface acidity.^[^ ^18^, ^19^, ^20^ ^]^ In part, this was based on their inability to detect corresponding changes in the electrical conductivity of their films and little detectable change in the oxidation state of the mixed valent elements in the MIEC surface. We suspect that the smaller changes in conductivity compared to that of *k*_chem_ were primarily due to the bulk‐dominated character of conventional MIEC electrodes.^[^ ^26^, ^27^, ^28^ ^]^ Given the nanometer‐scale dimensions of space charge regions and the low surface coverage (1.5–3%) achieved during the infiltration process, the influence of acidity‐controlled heterointerfaces on overall conductivity was limited.^[^ ^22^, ^29^ ^]^ These issues, therefore, necessitate the development of an alternative platform with a much higher surface‐to‐volume ratio to maximize the influence of surface conduction and examine precisely the impact of heterointerface acidity‐mediated space charge on the near‐surface electronic properties. Such an approach should serve to unveil the correlation between modified local space charge potentials induced by surface acidity and the corresponding electrical conductivity changes.

In this study, we successfully demonstrate the marked effects of heterointerface acidity on the electronic properties of an optimized nanostructured platform composed of well‐aligned Pr_0.2_Ce_0.8_O_2‐δ_ (PCO20) nanowire arrays (PCO_NA_). The conductivity of PCO_NA_ is demonstrated to vary by three orders of magnitude over the temperature range of 450–650 °C, following changes in relative Smith acidity of infiltrated binary oxides, ranging from basic (Li_2_O) to acidic (SiO_2_). Moreover, serial infiltration with Li_2_O following reductions in electrical conductivity induced initially by SiO_2_, not only recovers, but exceeds the conductivity initially measured by nearly the same factor. We also report, for the first time, and consistent with the above observations, that the relative acidity of insulating substrates (Al_2_O_3_ vs MgO) supporting PCO_NA_ can also significantly impact its electronic properties. Finally, we note that in polycrystalline materials, space charge potentials at grain boundaries (GBs) must also be accounted for in rationalizing conductivity changes, along with those associated with the heterointerfaces. This is because cation diffusion from substrates or surface infiltrants into polycrystalline thin films can alter their GB space charge potentials, depending on whether in‐diffused species act as donors or acceptors at the specific GB lattice sites they occupy. As a result, the overall conduction pathway governing the electronic properties of PCO_NA_ is determined by the acidity‐mediated space charge potentials at both the heterointerfaces and GBs, representing clear and strong evidence for the critical role of local space charges in MIEC electrodes.

## 2 Results and Discussion

### 2.1 Fabrication and Characterization of PCONA as a Model Platform

**Figure 1:**
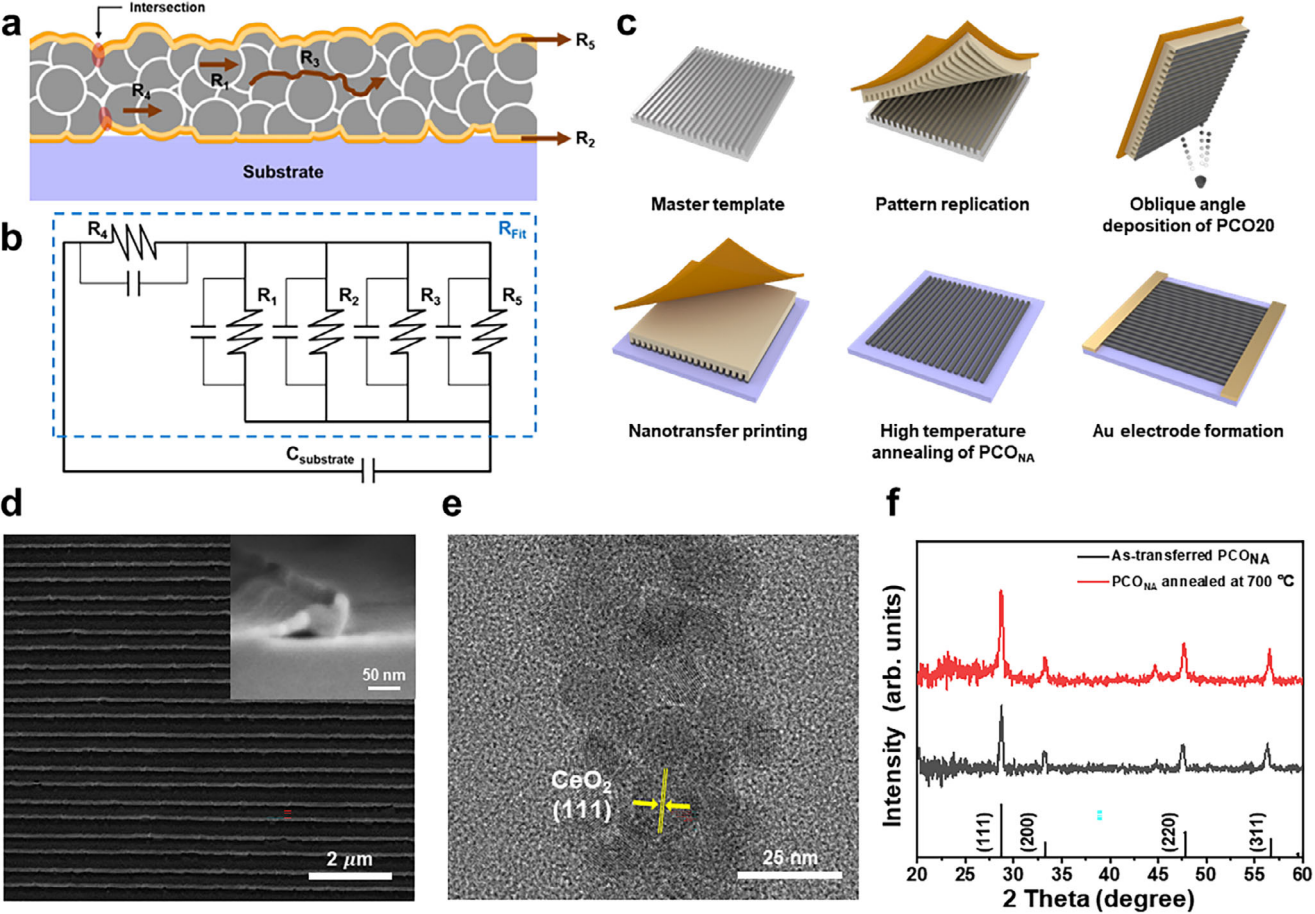
Fabrication and characterization of PCO20 nanowire arrays (PCO_NA_) as a model platform for analyzing effects of heterointerface acidity on electronic properties. a) Schematic cross‐sectional illustration of several conduction pathways in PCO nanowires. The outer orange lines mark the heterointerfaces while the inner yellow lines aid in visualizing the regions within the grains influenced by the relative acidity of the surface infiltrants or substrate. The transparent oblong red circles at the left indicate the regions where the heterointerface and grain boundary interact. b) The corresponding conceptual equivalent circuit represents how the electrical conduction pathways illustrated in (a) are interconnected. c) Fabrication process of PCO_NA_ model platform through solvent‐assisted nanotransfer printing method with pulsed laser deposition (PLD) and high‐temperature annealing. d) SEM image of fabricated PCO_NA_ (inset image: Cross‐sectional SEM image of fabricated PCO_NA_) and e) TEM image of fabricated PCO nanowire. f) XRD patterns of as‐transferred PCO_NA_ and fabricated PCO_NA_ after high‐temperature annealing at 700 °C. Black vertical lines at the bottom of the figure indicate the dominant facets of the cerium oxide crystal structure.

**Figure** **1**a illustrates a cross‐sectional view of a PCO_NA_ model platform supported on an insulating substrate to elucidate the role of local space charges induced by modulated surface or substrate acidity on electronic properties. In addition to the bulk conduction (*R*_1_), four other possible conduction pathways within PCO_NA_ have been depicted as conduction through substrate heterointerfaces (*R*_2_), along GBs (*R*_3_), perpendicular to GBs (*R*_4_), and surface heterointerfaces with infiltrants (*R*_5_). Because space charge potentials at heterointerfaces and GBs are affected by the relative acidity of binary oxides and the in‐diffusion of cations along GBs sourced from the binary oxides, all these conduction pathways need to be considered. Notably, all other conduction pathways are always in series with conduction perpendicular to the GBs (*R*_4_), so the overall equivalent circuit can be described as shown in Figure 1b.

To achieve a high surface‐to‐volume ratio to enhance the relative ratio of surface to bulk conduction, PCO_NA_ was fabricated using a solvent‐assisted nanotransfer printing method (S‐nTP) (Figure 1c), previously reported.^[^ ^30^, ^31^, ^32^ ^]^ This morphology offers readily accessible open structures for the conformal formation of interfaces with both substrates and infiltrants. As shown in the scanning electron microscopy (SEM) images (Figure 1d), PCO_NA_ with widths of 50 nm, a pitch of 150 nm, and a thickness of 50 nm was fabricated on insulating substrates.

Due to the shadowing effect caused by oblique angle deposition,^[^ ^33^ ^]^ Pr_0.2_Ce_0.8_O_2‐δ_ (PCO20) was deposited only on one side of the PMMA, resulting in an unconventional droplet‐shaped structure with a tail‐like cross‐section (see inset and Figure S1, Supporting Information). This nanoscale morphology contributes to an increased surface‐to‐volume ratio, intensifying the influence of surface properties compared to that of the bulk, and provides a large number of parallel conductive nanowire channels (estimated to be 50 000) for precise analysis of the in‐plane electrical conductivity. The concentration of Pr (20 at%) in the fabricated PCO_NA_ was identical to that of the target source as confirmed by inductively‐coupled plasma mass spectrometry (ICP‐MS) (Table S1, Supporting Information). The accompanying transmission electron microscopy (TEM) image shows that the PCO20 nanowires are composed of nanoscale grains with diameters ranging from a few up to 20 nm (Figure 1e), and that these nanosized grains result in a rough surface, which amplifies the impact of the surface on properties. While PCO20 is shown to be polycrystalline, most of the crystal planes are observed to consist of CeO_2_ (111) planes with d‐spacings of 0.31 nm. X‐ray diffraction (XRD) patterns also assign the main peak to the (111) plane, although other oriented planes also exist (Figure 1f). Furthermore, the identical positions of peaks appearing in fabricated PCO_NA_ compared to as‐transferred samples imply that strain effects are negligible.^[^ ^15^, ^34^ ^]^ X‐ray photoelectron spectroscopy (XPS) shows only peaks associated with Ce/Pr, with no evidence of other species such as Sr and Cr which would cause degradation as shown in Figure S2 (Supporting Information). Finally, Au current collectors were sequentially deposited on both ends of the PCO_NA_ using Au paste for evaluating in‐plane conductivity. The distance between the two current collectors and the width of the model platform were fixed to 1 mm and 1 cm, respectively.

### 2.2 Surface‐Infiltrated Oxide Acidity Effects on PCONA Electronic Properties

To investigate the effects of binary oxide acidity on the electrical conductivity of PCO_NA_, two PCO_NA_ specimens with different binary oxides infiltrated onto their surfaces were prepared and deposited on single‐crystal sapphire (Al_2_O_3_) substrates. The in‐plane conductivity of uninfiltrated PCO_NA_ as well as PCO_NA_ infiltrated by SiO_2_ (acidic) and by Li_2_O (basic) was measured utilizing AC impedance spectroscopy, respectively. Additionally, to explore the potential for recovering degraded conductivity, PCO_NA_ with serial infiltration of Si‐species followed by Li‐species was also prepared and evaluated.^[^ ^25^ ^]^

The primary objective of this study was to monitor conductivity changes induced by heterointerfaces through the surface infiltration of binary oxides with different acidity, rather than the initial focus on the oxygen exchange rate as reported previously.^[^ ^22^, ^35^, ^36^ ^]^ Hence, we attempted to achieve full PCO_NA_ coverage by using a concentrated solution (0.2 m) to maximize coverage and as shown in SEM images in Figure S3 (Supporting Information), the coverage is complete. It is important to note that the electrical conductivities of the insulating binary oxide infiltrants used in this study are negligible compared to that of the PCO nanowires (Table S2, Supporting Information). The measured impedance spectra are therefore assumed to solely represent PCO_NA_ electrical pathways. AC impedance spectra were obtained between 450–650 °C to extract the in‐plane conductivity of both the uninfiltrated and infiltrated samples (Figure S4, Supporting Information).

**Figure 2:**
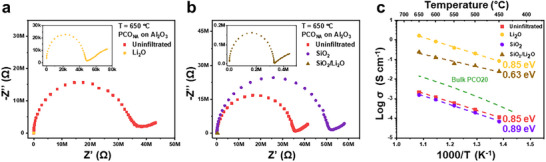
Influence of infiltrated binary oxide acidity on PCO_NA_ electronic properties. a) Impedance spectra of pristine PCO_NA_ followed by Li‐infiltration at 650 °C under a *pO_2_* of 0.21 atm (see inset for reduced resistance). b) Impedance spectra of pristine PCO_NA_ followed by Si‐infiltration and serial Li‐infiltration for recovering conductance (see inset for reduced resistance) at 650 °C under a *pO_2_* of 0.21 atm. Al_2_O_3_ used as a substrate in both cases. c) Arrhenius plots of in‐plane conductivity of pristine PCO_NA_ followed by Li‐, Si‐, and serial Li‐infiltrations along with their respective activation energies, respectively. The green dashed line indicates the conductivity behavior of a bulk PCO20 film calculated based on previously reported defect chemical and kinetic modelling.^[^ ^37^ ^]^

As illustrated in **Figure** **2**a,b, Li‐infiltration resulted in nearly three orders of magnitude decrease of resistance (see inset), while Si‐infiltration caused only a slight increase in resistance, both relative to the uninfiltrated PCO_NA_. Arrhenius plots of in‐plane conductivity under each infiltration condition, along with the corresponding activation energy (*E_a_*) values, are summarized in Figure 2c. Consistent with the impedance plots, Li‐infiltrated PCO_NA_ exhibited approximately three orders of magnitude higher in‐plane conductivity compared to uninfiltrated PCO_NA_ over the full temperature range, with the same nominal *E_a_* of 0.85 ± 0.023 eV. However, only 1.5 times lower conductivity was measured in Si‐infiltrated PCO_NA_ relative to the uninfiltrated reference with similar *E_a_* (0.89 ± 0.025 eV). A similar trend was also observed in serial infiltration with Li‐species following Si infiltration, aimed at recovering conductivity, as shown in Figure 2b (see also inset). Subsequent Li infiltration on Si‐infiltrated PCO_NA_ not only restored, but exceeded the initial conductivity by 100‐fold with a reduced *E_a_* of 0.63 ± 0.065 eV. Although serial Li infiltration did not achieve as much improvement in conductance as sole Li infiltration, given the pre‐coating effects of SiO_2_, it once again verified the strong positive impact of basicity on already degraded electrodes.

### 2.3 Insulating Substrate Acidity Effects on PCONA Electronic Properties

We next explored the impact of the choice of insulating substrate on the electronic properties of our PCO_NA_. This is not only of relevance to further validate the observed trends of heterointerface acidity on the electrical conductivity of PCO_NA_, but also considering that diverse insulating substrates, such as Al_2_O_3_ or MgO, are commonly used to study the electrical properties of deposited oxide thin films. Generally, one employs such substrates to support controlled film growth while minimizing the potential for short‐circuiting of the supported films through the substrate. The potential impact of their interfacial properties on the overlaying films’ conductivity, however, is generally ignored based on the assumption that transport along such interfaces would have an insignificant impact compared to transport through the bulk (*R*_1_). It is noteworthy that most of these insulating substrates each possess their own distinct relative Smith acidities, and thereby work functions, which could presumably result in inducing differing space charge phenomena at these interfaces.^[^ ^38^ ^]^ PCO_NA_ were fabricated on both Al_2_O_3_ and MgO substrates (Figure S5, Supporting Information), expected to exhibit acidic and basic characteristics respectively relative to PCO20. This enabled us to assess any alterations in the in‐plane conductivity induced at the heterointerface between the substrates and PCO_NA_.

**Figure 3:**
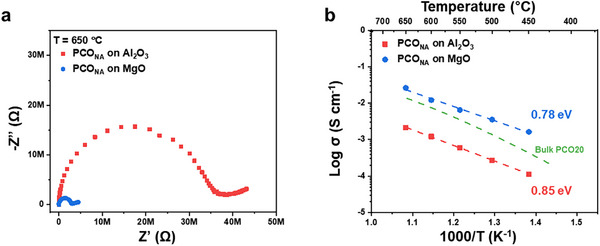
Influence of insulating substrate acidity on PCO_NA_ electronic properties. a) Comparison of impedance spectra of PCO_NA_ fabricated on Al_2_O_3_ and MgO substrates measured at 650 °C under a *pO_2_* of 0.21 atm. b) Arrhenius plots of in‐plane conductivity of PCO_NA_ fabricated on Al_2_O_3_ and MgO substrates along with their respective activation energies. The green dashed line indicates the conductivity behavior of a bulk PCO20 film calculated based on previously reported defect chemical and kinetic modelling.^[^ ^37^ ^]^

The AC impedance spectra of PCO_NA_ fabricated on the two different substrates were obtained for temperatures between 450–650 °C as shown in Figures S4,S6 (Supporting Information). By comparison of these two impedance spectra at the same measurement temperature of 650 °C (**Figure** **3**a), it becomes immediately obvious that PCO_NA_ fabricated on Al_2_O_3_ is over an order of magnitude more resistive than the same structure fabricated on MgO. Arrhenius plots of in‐plane conductivity are shown in Figure 3b along with a listing of their respective *E_a_* values. Consistent with the impedance plots, PCO_NA_ on the MgO substrate exhibited ≈10 times higher in‐plane conductivity compared to PCO_NA_ on the Al_2_O_3_ substrate over the full temperature range, with a nominally lower *E_a_* of 0.78 ± 0.037 versus 0.85 ± 0.007 eV. Since all other conditions were identical except for the choice of substrate, it is reasonable to assume that the differences in conductivity between these two samples can be attributed to the relative acidity of the substrates. With this insight, it can be argued that the selection of a suitable substrate must be considered before depositing nanostructured semiconductors or MIECs to avoid such heterointerface acidity effects. Indeed, we suspect that many reports of thin film oxide conductivity have been impacted by the relative substrate acidity of the supporting substrate and thus need to be reevaluated or appropriately normalized (Note S5, Supporting Information).

### 2.4 Acidity‐Mediated Space Charge Potential at Heterointerfaces and GBs

**Figure 4:**
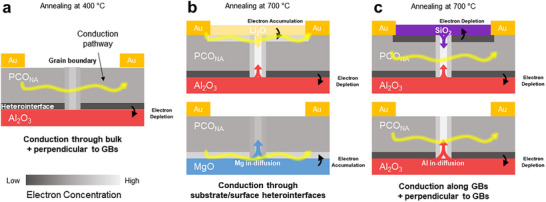
Schematic illustration of heterointerface acidity‐mediated space charge potentials at heterointerfaces and GBs of PCO_NA_, and impact of electron redistribution a) PCO_NA_ annealed at 400 °C. Conduction mainly occurs through bulk (*R*_1_) and perpendicular to GBs (*R*_4_). The substrate heterointerface shown in black corresponds to electron depletion due to the relative acidity of Al_2_O_3_. Slightly brighter GB is attributed to a built‐in space charge potential (positive core charge) leading to some electron accumulation. For high‐temperature annealing, cation diffusion into GBs must be considered. Diffusion of cations up to GBs leads to GB electron accumulation/depletion, described in white/gray (see (b) and (c)). b) Li‐infiltrated PCO_NA_ fabricated on Al_2_O_3_ substrate (upper image) and PCO_NA_ fabricated on MgO substrate (lower image) annealed at 700 °C. Conduction mainly occurs through substrate (*R*_2_) (upper image) or surface (*R*_5_) (lower image) heterointerfaces. Substrate/surface heterointerface shown in light grey corresponds to electron accumulation due to the relative basicity of Li_2_O and MgO. c) Si‐infiltrated PCO_NA_ (upper image) and PCO_NA_ fabricated on Al_2_O_3_ substrate (lower image) annealed at 700 °C. Conduction mainly occurs along GBs (*R*_3_) and perpendicular to GBs (*R*_4_). Substrate/surface heterointerface shown in black corresponds to electron depletion due to the relative acidity of SiO_2_ and Al_2_O_3_. The scale of electron concentration is represented through the color bar at the bottom left.

Based on the relative acidity of infiltrants or substrates relative to PCO, adjacent acidic/basic species were expected to induce electron depletion/accumulation at the heterointerfaces with PCO_NA_, thereby leading to increased/decreased resistance along both the conduction pathways through substrate (*R*_2_) and surface (*R*_5_) heterointerfaces. As we have shown in Figures 2c and 3b, elements with higher acidity relative to PCO (Al_2_O_3_ and SiO_2_) resulted in decreases in conductivity, while elements with lower acidity relative to PCO (MgO and Li_2_O) resulted in increases in conductivity, consistent with our expectation. However, several details need to be explained in more detail to fully appreciate the observed trends:

I) The relative magnitude of change in the conductivity of PCO_NA_ driven by surface acidity modulation with basic and acidic elements compared to uninfiltrated samples differed significantly as illustrated in Figure 2c,b. This divergence might originate from the distinct conduction pathways determined by space charge potentials at the heterointerfaces. For example, in the case of acidic treatments, the higher relative acidity compared to that of PCO is expected to generate a more resistive conduction pathway through the surface/substrate heterointerfaces (*R*_5_/*R*_2_), leading to the expectation that conduction occurs primarily through the bulk (*R*_1_) (**Figure** **4**a,c). On the other hand, the treatment with basic elements will result in carrier enhancement due to electron accumulation and thereby enhanced conduction through the surface/substrate heterointerface (*R*_5_/*R*_2_) (Figure 4b). Interestingly, Li‐infiltration had a much larger impact (three orders of magnitude at 450 °C) on the PCO_NA_ conductivity than the MgO substrate (factor of ≈10 at 450 °C), although both have a higher basicity than PCO. This can be related to i) the very different natures of the two interfaces (the unusual droplet‐shape of the top rough surface of PCO_NA_ leads to a much higher interface area than at the planar substrate interface) and ii) the higher relative basicity of Li_2_O is expected to result in higher electron accumulation and therefore higher conductivity compared to MgO.

II) In the case of acidic treatment with SiO_2_ infiltration or use of Al_2_O_3_ substrate, one would expect, as mentioned above, the conductivity to solely conduct through the bulk (*R*_1_), and therefore for it to reach similar values to that of the bulk PCO20 reference. However, the conductivity and its *E_a_* for PCO_NA_ are reduced in both cases compared to bulk PCO20 (1 eV), which indicates that conduction is impacted by pathways in addition to the bulk conduction (*R*_1_). Here, one must consider how grain boundaries (GBs), as well, can potentially serve as either short‐circuiting pathways (*R*_3_) or serve to block transport (*R*_4_), as illustrated in Figure 1a,b. While we do not report any direct measurements of GB characteristics in this study, based on their expected behavior, we can explain the above observations. In the following, we therefore briefly review key relevant findings describing the roles of GBs in impacting charge transport in ceria‐based systems and identify how small polaron hopping mobility, characteristic of electron transport in PCO, can result in GB phenomena contrary to normal expectations. For those interested, more detailed discussions of these issues may be found in Note S2 (Supporting Information).

GBs in ceria systems,^[^ ^39^, ^40^ ^]^ in general, exhibit a positive space charge potential associated with a positive GB core charge, causing a depletion of oxygen vacancies and accumulation of electrons in the adjacent space charge zones.^[^ ^41^ ^]^ In nominally undoped CeO_2_, the accumulation of electrons has been shown to lead to electronically conducting layers that bypass bulk ionic transport, thereby controlling the overall conductivity.^[^ ^42^, ^43^, ^44^ ^]^ On the other hand, as mentioned above, this same positive GB space charge potential in acceptor‐doped CeO_2_ (e.g., Gd‐doped CeO_2_ – GDC), an oxygen ion conductor, can lead to orders of magnitude reduction in oxygen ion conduction due to oxygen vacancy depletion at the GBs.^[^ ^39^, ^45^ ^]^ As recently demonstrated by the authors, this space charge potential can be tuned by in‐diffusion of substrate elements (i.e., Mg and Al) into GDC thin films at intermediate temperatures (700–900 °C),^[^ ^45^ ^]^ as described in Note S2 (Supporting Information). We suspect correspondingly significant Al up‐diffusion occurred along GBs in PCO_NA_ samples grown on alumina substrates annealed at 700 °C (Figure 4c, lower image), compared to those annealed at 400 °C (Figure 4a), leading to an increase of the positive space charge potential at the GBs (Figure 4c). While conduction along the GBs (*R*_3_), in principle, should give enhanced conduction due to accumulation of electrons around GBs, that transport path nevertheless would have to pass through a mobility blockage related to the small polaron hopping controlling electronic transport in PCO connected with the path perpendicular to GBs (*R*_4_), as discussed in Note S2 (Supporting Information) and displayed in Figures S7,S8 (Supporting Information). This results in reduced *E_a_*, attributed to the enhanced space charge potential at the GBs, yet without a corresponding increase in conductivity. In fact, it is even lower than that of the PCO_NA_ annealed at 400 °C (Figure S9, Supporting Information), where Al up‐diffusion is expected to be kinetically restricted and therefore the space charge potentials at the grain boundaries are lower. The lower conductivity is related to the inability of electrons to hop from occupied Pr^3+^ sites to adjacent unoccupied Pr^4+^ sites under conditions of high electron accumulation at the GBs. XRD patterns showed no difference in peak position and shape of PCO_NA_ annealed at different temperatures suggesting that no differences in strain were present (Figure S10, Supporting Information). On the other hand, in the case of the nanowires sitting on MgO, transport across GBs (*R*_4_) becomes less resistive due to the up‐diffusion of Mg at the GBs, lowering the space charge potential, allowing for the measurement of the un‐impeded conductivity enhancement parallel to the substrate heterointerface (*R*_2_) (Figure 4b). Thus, the similarities in *E_a_* between the PCO_NA_ treated with acids and bases, but different conductivity trends, originate from the presence of positive space charge potential at different dominant conduction pathways.

Interestingly, opposite trends were observed in a purely ionic conducting ceria system, 3 mol% Gd‐doped ceria (GDC3), prepared via the same method as PCO_NA_ (Note S3, Supporting Information). Here, the basic treatment with MgO as a substrate leads to a total decrease in conductivity in contrast to Al_2_O_3_. This observation aligns very well with the space charge model, as the majority of carriers in this case are positively charged oxygen vacancies ($V_{o}^{.}$), and therefore the space charges present at the heterointerface are expected to impact the carrier in the opposite direction to the electronic carriers in PCO_NA_. The activation energies measured in the GDC3 nanowire arrays are characteristic of grain boundary‐controlled resistance and are clearly influenced by the substrate heterointerfaces, similar to the case of the PCO_NA_ (Figure S11, Supporting Information). Therefore, the Smith acidity scale is a universally applicable descriptor for predicting the influence of heterointerface space charge effects not only on electronic but also ionic carriers.

III) Finally, the relatively moderate enhancement achieved in sequential infiltration with Li‐species following Si (Figure 2c), compared to solely Li‐infiltrated PCO_NA_, can be attributed to the effective Smith acidity of Li–Si compounds.^[^ ^46^ ^]^ As evidenced by the XPS spectra (Figure S12, Supporting Information), Li‐species react with SiO_2_ at high temperatures, forming intermediate lithium silicate composites (Li_2_SiO_3_ or Li_4_SiO_4_).^[^ ^47^ ^]^ The acidity of these composites should be located between the Smith acidity scale of Li_2_O and SiO_2_, which can be expressed as:

(1) $$a_{Li_{2}\mathrm{Si}O_{3}}=\frac{2a_{Li_{2}O}+a_{\mathrm{Si}O_{2}}}{3}\;\;or\;\;a_{Li_{4}\mathrm{Si}O_{4}}=\frac{4a_{Li_{2}O}+a_{\mathrm{Si}O_{2}}}{5}\;$$

Due to the higher basicity of Li_2_O and its dominant influence, lithium silicate exhibits strong basicity, in contrast to SiO_2_, and plays a role in accumulating electrons at the top heterointerface of PCO_NA_, although obviously not as high as Li_2_O alone. This leads to the formation of a dominant conduction pathway through the surface heterointerface (*R*_5_). This change in effective Smith acidity leads to a 100‐fold improvement in conductivity.

We suggest that frameworks driven by the Smith acidity scale enable us to predict the space charge properties of oxide‐based heterointerfaces. One can therefore expect application of Smith acidity criteria in predicting heterointerface properties to have a very broad impact given that they can be readily applied not only to MIECs, demonstrated by Steinbach et al., but also to semiconducting and even insulating oxides. This makes the approach suitable for application to a much wider temperature range of devices even for microelectronics operating at room temperature. Furthermore, the proposed framework achieved both enhancement and suppression of space charge potential depending on the relative surface acidity. By demonstration and modeling this approach with polycrystalline PCO_NA_ rather than single‐crystal samples, it was possible to successfully account for charge transport behavior not only across but also along the heterointerfaces and GBs. This served to provide more realistic insights into acidity‐mediated space charge engineered conductivity, with significant implications for practical nanostructured thin film applications.

## 3 Conclusion

In summary, we have investigated how the PCO_NA_ model platform enables direct observation of orders of magnitude conductivity changes induced at the respective heterointerfaces and GBs, confirming the influence and significance of surface acidity‐mediated local space charge potentials. These correlations were firmly established by examining the electronic properties of PCO_NA_ with different infiltrants, insulating substrates, and annealing temperatures. Notably, a single Li‐infiltration led to a remarkable three orders of magnitude increase in conductivity. Moreover, our findings challenge the assumption in many studies that insulating substrates do not affect electrical measurements, as their relative acidity can significantly impact measured conductivity at the film substrate interface and upon higher temperature anneals, impact film GB properties as well. Furthermore, our study highlights how the in‐diffusion of heterointerface elements at film GBs can also markedly modify the GB transport properties, which need to be taken into account to fully understand the total changes in conductivity of such nanocrystalline thin films. These features play a direct and critical role in determining the electronic properties of nanostructured functional oxides. This strongly supports the concept of the Smith acidity scale as a predictive descriptor for space charge behavior at heterointerfaces in functional and electrocatalytically active oxide systems. Our understanding of the possible role of supporting substrate in modulating the electronic properties of thin oxide films opens up new opportunities for the modulation of thin film properties without altering their defect chemistry, oxygen stoichiometry, or microstructure.

## 4 Experimental Section

### Preparation of Pr_0.2_Ce_0.8_O_2‐δ_ (PCO20) Target Source for Pulsed Laser Deposition

The PCO20 oxide target was synthesized through a sol–gel method, involving dissolving cerium and praseodymium nitrates with chelating agents in water, adjusting the pH to 9.5, and heating at 80 °C to induce gelation. The gel was dried, fired at 450 °C, calcined at 750 °C to produce PCO20 powder, which was then pelletized and sintered at 1400 °C to form the pulsed laser deposition target.

### Fabrication of Pr_0.2_Ce_0.8_O_2‐δ_ Nanowire Arrays (PCONA) on Insulating Substrates

PCO_NA_ were fabricated using a combination of the solvent‐assisted nanotransfer printing (S‐nTP) method and PLD, as illustrated in Figure 1a. Initially, a Si master mold with line‐patterned features (line width: 50 nm, line pitch: 150 nm) was prepared via KrF photolithography and reactive ion etching. To facilitate easy detachment of the polymer transfer medium, a layer of polydimethylsiloxane (PDMS, Polymer Source Inc.) was applied to the Si master mold. Subsequently, a solution of polymethylmethacrylate (PMMA, Sigma‐Aldrich Inc.) dissolved in a mixed solvent of toluene, acetone, and heptane was spin‐cast onto the pre‐treated Si master mold to create the polymer transfer medium. The reason for mixing those three solvents was to optimize the surface energy of the layer with respect to the substrate. The PMMA transfer medium was then peeled off from the Si master mold using a polyimide adhesive tape (PI, 3 m Inc.), resulting in a reverse morphology of the Si master mold shape. Pr_0.2_Ce_0.8_O_2‐δ_ was deposited using PLD with an oblique‐angle of 80° to guide deposition selectively onto the sections that protrude from the PMMA transfer medium. PLD (Coherent COMPex Pro 205) was performed using a 248 nm KrF excimer laser operating at an energy level of 300 mJ under a frequency of 10 Hz at room temperature. Following deposition, discrete PCO_NA_ were obtained. PCO_NA_ were transfer‐printed onto target substrates (Al_2_O_3_ and MgO with a thickness of 0.5 mm), and the PMMA was removed via washing with toluene or acetone. Due to the limitations imposed by the PMMA transfer medium, high‐temperature deposition for improving crystallinity during PLD was not feasible. Thus, the resulting PCO_NA_ on the target substrates was annealed at 700 °C under ambient atmosphere for 2 h.

### Physicochemical Characterization of Materials

The physical and chemical characterization of PCO_NA_ involved a range of techniques including scanning electron microscopy (SEM), transmission electron microscopy (TEM), X‐ray diffraction (XRD), X‐ray photoelectron spectroscopy (XPS), and inductively coupled plasma mass spectrometry (ICP‐MS). SEM analysis was performed using a Hitachi S‐4800 with an acceleration voltage of 10 kV, while TEM imaging was conducted with FEI Tecnai G2 F30 S‐Twin operating at an acceleration voltage of 300 kV. High‐angle annular dark‐field scanning TEM, selected area electron diffraction pattern, and energy dispersive X‐ray spectrometer mapping were obtained using a TEM (JEOL, JEM‐2100F HR) operated at an acceleration voltage of 200 kV. Crystal information of the PCO_NA_ was determined through XRD (RIGAKU, SmartLab) conducted in *θ*–2*θ* scan mode with a Cu Kα1 incident beam. While the model platform used in this study consists of a single layer of PCO_NA_, the large void spaces between nanowires and the limited absolute amounts of materials make it unsuitable for obtaining a clear XRD diffraction pattern. Therefore, the XRD patterns were obtained by using 15‐layer stacked samples for reliable measurement. XPS (Thermo VG Scientific, K‐Alpha) measurements provided insights into the chemical compositions, oxidation states, and charge transfer evidence, with the C 1*s* peak at 284.8 eV serving as the reference for binding energy calibration. ICP‐MS (Agilent, ICP‐MS 7700S) was employed to analyze the chemical composition of PCO_NA_, with experiments repeated at least five times to ensure accuracy.

### Electrical Conductivity Measurements

The geometric factors associated with the nanowire‐formed electrically conductive channels assured that their overall resistance was significantly larger than that of the electrode contact resistance. Thus, the electrical properties measured could be predominantly attributed to the PCO_NA_. Electrical conductivity assessments of the PCO_NA_ were conducted using a two‐probe electrode system. AC impedance spectroscopy was carried out using a Solartron 1255 HF frequency response analyzer and EG&G PAR potentiostat across a frequency range of 0.1 to 100 000 Hz, within a temperature range of 450–650 °C (at intervals of 50 °C), under varying oxygen partial pressure (ranging from 0.1 to 1 atm). AC impedance spectroscopy was applied to ensure the ability to isolate the total nanowire conductivity (high frequency contribution) from the electrode (low frequency) contributions, and in‐plane conductivity measurements were made while lowering the temperature at each annealing temperature. All the impedance spectra were fit to an equivalent RC circuit because only a single large semicircle could be observed at higher frequencies in all data sets, that it was assigned to the total nanowire conductivity. While a small tail at lower frequencies was also present, this could be associated with the electrodes, not of interest for this analysis. The various individual contributions to the total conductivity of the nanowire array could not be further deconvoluted because they were shielded by the stray capacity that forms between the substrate and the electrodes. This was validated by comparing the measured capacitance, of ≈10^−11^ F for all data sets, independent of infiltration, with the expected substrate capacitance, ≈10^−11^ F, obtained by assuming a simple parallel plate capacitor, described by: $C=\varepsilon_{r}\;\varepsilon_{0}\left(\frac{LN}{d}\right)$, where L is the length of digits, N the number of pairs of digits, d the distance between digits, and ε_*r*_ε_0_ the dielectric constant of the materials. The reproducibility of the conductivity was confirmed by examining the impedance spectra obtained for three different uninfiltrated PCO_NA_ samples. The experimental setup involved placing the samples in an alumina tube, with a gold current collector linked to a gold wire, while gas mixtures of oxygen and nitrogen were regulated through digital mass flow controllers. In‐plane conductivity was monitored by recording voltage measurements every 0.5 s until equilibrium was reached using AC measurements. Initial conductivities of PCO_NA_ without infiltration were evaluated to establish reference values.

### Surface Infiltration of Binary Oxides on PCONA

The infiltration method involved a simple drop and drying process on PCO_NA_ with ethanol‐based solutions of tetraethylorthosilicate (TEOS) and Li(NO_3_)_3_. Solutions containing Li nitrate and TEOS at a concentration of 0.2 m were prepared for the infiltration process. Initial conductivities of PCO_NA_ without infiltration were evaluated to establish reference values. Infiltration was performed with the sample located in its original cell setup, ensuring complete solution coverage and drying of 50 µL, thereby enhancing infiltration reliability. These precursors decompose and convert to binary oxides (SiO_2_ and Li_2_O, respectively) at temperatures exceeding 600 °C under an air atmosphere.^[^ ^24^ ^]^ Thus, PCO_NA_ was annealed under air conditions at 650 °C and measurements initiated after stabilization of the conductivity was achieved.

## Conflict of Interest

The authors declare no competing interests.

## Author Contributions

G.R.L. and T.D. contributed equally to this work. G.R.L. and H.L.T. conceived the original project. G.R.L., T.D., H.G.S., and H.L.T. designed the experimental protocol. G.R.L. conducted the overall sample preparation, the physicochemical property characterization, and the electrochemical measurements. T.D. proposed the modeling of the electronic properties results. G.R.L. and T.D. analyzed the experimental and computational results. J.K. fabricated the target source for PLD and performed the deposition. H.L.T. supervised the project. G.R.L., T.D., Y.S.J., and H.L.T. wrote and reviewed the manuscript with input from all the authors. All authors discussed the results and contributed to revisions of the manuscript.

## Supporting information



Supporting Information

## Data Availability

The data that support the findings of this study are available from the corresponding author upon reasonable request.
