# Supplementary material for: Acidity‐Mediated Metal Oxide Heterointerfaces: Roles of Substrates and Surface Modification
Source: Adv Mater. 2025 Oct 4;38(3):e12804. doi: 10.1002/adma.202512804 (PMC12801377; doi:10.1002/adma.202512804)
Supplement: Supplementary file 1 — Supporting Information [file ADMA-38-e12804-s001.docx]

Supporting Information

Acidity-Mediated Metal Oxide Heterointerfaces: Roles of Substrates and Surface Modification

*Gyu Rac Lee, Thomas Defferriere, Jinwook Kim, Han Gil Seo, Yeon Sik Jung, and Harry L. Tuller*^*^

G. R. Lee, T. Defferriere, and H. L. Tuller

Department of Materials Science and Engineering, Massachusetts Institute of Technology, Cambridge, MA 02139, USA

E-mail: hltuller@mit.edu

J. Kim

Department of Materials Science and Engineering, Northwestern University, Evanston, IL 60208, USA

H. G. Seo

Department of Materials Science and Engineering, Dankook University, 119 Dandae-ro, Dongnam-gu, Cheonan-si, Chungnam 31116, Republic of Korea

Y. S. Jung

Department of Materials Science and Engineering, Korea Advanced Institute of Science and Technology, 291 Daehak-ro, Yuseong-gu, Daejeon 34141, Republic of Korea

**The supplementary materials include:**

Note S1 to S5

Table S1, S2

Figure S1 to Figure S13

**Note S1. Fabrication of PCO_NA_ by solvent-assisted nanotransfer printing combined with pulsed laser deposition**

Pr_0.2_Ce_0.8_O_2-δ_ (PCO20) was selected as a model MIEC oxide due to its high chemical stability,^[1]^ free of elements that tend to segregate to the surface, for example Sr in LSCF and STF,^[2–4]^ and the dominance of electronic over ionic conductivity at high pO_2_ (e.g. in this study in air).^[5,6]^ In conventional solvent-assisted nanotransfer printing (S-nTP), electron beam evaporation is typically used to deposit the target materials, however, this is not suitable for depositing complex oxide materials normally used as MIEC electrodes such as PCO20. Electron beam irradiation, when applied to metal oxides, leads to the reduction and dissociation of the target source into metal and metal suboxide species, each with different volatilities, thereby hindering the reproduction of the stoichiometry of the target source in the deposited film.^[7,8]^ Pulsed laser deposition (PLD) was therefore selected as an alternative to electron beam evaporation, given its ability to reproduce films with nearly the same stoichiometry as the target source.^[9]^ It is worth noting that this strategy is not limited to PCO used in this study but is extendable to other materials that can be deposited via PLD. We believe that our strategy exhibits wide-ranging applicability and potential for elucidating the precise role of acidity-mediated local space charge across various materials and applications.

**Note S2. Modeling of heterointerface acidity-mediated space charge effects on electronic properties of PCO_NA_**

Zhang et al. previously showed that decreasing the grain size of a bulk PCO20 sample by lowering the sintering temperature led to a corresponding decrease in conductivity and in *E_a_* from 1 eV down to 0.65 eV. They attributed this to increased strain, that lowers the reduction enthalpies, increases the $\left[ {Pr}^{3+} \right]$ concentration, and therefore reduces the conductivity by decreasing the availability of small polaron hopping sites.^[10]^ As we show in the Arrhenius plot of **Figure S9**, PCO_NA_ prepared on an Al_2_O_3_ substrate and annealed at 400 °C instead of 700 °C, exhibited similar *E_a_* and magnitude of conductivity comparable to that of bulk PCO20, despite the sample being composed of nanoscale grains that are presumably smaller due to the lower annealing temperatures.

To rationalize our observations, we note that the activation energy of conductivity (*E­_a_*) in bulk PCO is reportedly made up of two terms, given that the electron concentration and mobility are both thermally activated. Following previous derivations,^[10]^ $\left[ {Pr}_{Ce}^{x} \right]$~ $\left[ {Pr}_{Ce,tot} \right]$ is assumed in the high pO_2_ regime where overall charge balance is assumed to be $\left[ {Pr}_{Ce}^{'} \right] \sim2[V_{O}^{..}]$. Then the small polaron electronic conductivity associated with ${Pr}^{3+}$ can be written as:

$\sigma_{{Pr}^{3+}=}{2^{\frac{1}{3}}\left[ {Pr}_{Ce,tot} \right]^{\frac{2}{3}}\left[ O_{O}^{x} \right]^{\frac{1}{3}}{K_{r,Pr}^{0}}^{\frac{1}{3}}\mu}_{Pr,0}\exp\left( -\frac{\frac{\Delta H_{r,Pr}}{3}+E_{m,Pr}}{k.T} \right)P_{O_{2}}^{-\frac{1}{6}}$ (1)

where $\Delta H_{r,Pr}$ is the reduction enthalpy of Pr and $E_{m,Pr}$ is the small polaron migration energy. Based on previous literature values, the expected bulk activation energy at high pO_2_ and low temperatures is therefore expected to be equal to $\frac{\Delta H_{r,Pr}}{3}+E_{m,Pr}$~ 1 eV, consistent with our results for PCO_NA_ fabricated on Al_2_O_3_ substrate annealed at 400 °C (**Figure S9**), where little impact from GB space charge effects was anticipated.

On the other hand, the carrier concentration is also expected to vary spatially according to the magnitude and polarity of the space charge potentials at both heterointerfaces and GBs. Since small polarons are mobile, they can readily redistribute within the space charge region in response to changes in space charge potential, similarly to the oxygen vacancies, a situation that can be described by the Gouy-Chapman approximation,^[11]^ where a flat spatial profile of [Pr_tot_] is assumed. To a first-order approximation, the variation in ${Pr}_{Ce}^{'}$ adjacent to the interface in response to the space charge potential profile can be defined as^[12]^ :

$\frac{\left[ {Pr}_{Ce}^{'} \right]\left( x \right)}{\left[ {Pr}_{Ce}^{'} \right]_{bulk}}=\exp\left( -\frac{ze}{kT}\Delta\varphi\left( x \right) \right)$ (2)

where the potential distribution $\Delta\varphi\left( x \right)$, can be obtained by solving the Poisson equation to account for the total electroneutrality between the interface and the space charge layer.^[12]^ An analytical solution is generally possible by making the depletion approximation, whereby the concentration of one of the carriers is negligible compared to the other (generally true when the two major carriers are of opposite sign, causing the depletion of one in the space charge region and the accumulation of the other).

In general, we can then define conductance parallel and perpendicular to the interface as:

$\sigma_{//,sc}=\frac{1}{L_{D}}\int_{0}^{2L_{D}} \left[ {Pr}^{3+} \right]\left( x \right)\left( 1-\frac{\left[ {Pr}^{3+} \right]\left( x \right)}{\left[ {Pr}_{tot} \right]} \right)q\mu_{Pr}dx$ (3)

$\rho_{\perp,sc}=\frac{1}{L_{D}}\int_{0}^{{2L}_{D}} \frac{1}{[{Pr}^{3+} ]\left( x \right)\left( 1-\frac{\left[ {Pr}^{3+} \right]\left( x \right)}{\left[ {Pr}_{tot} \right]} \right)q\mu_{Pr}}dx$ (4)

where $L_{D}=\left( \frac{kT\varepsilon}{2e^{2}\left[ {Pr}_{Ce}^{'} \right]_{bulk}} \right)^{0.5}$is the Debye length and $\left( 1-\frac{\left[ {Pr}^{3+} \right]\left( x \right)}{\left[ {Pr}_{tot} \right]} \right)=\frac{\left[ {Pr}^{4+} \right]\left( x \right)}{\left[ {Pr}_{tot} \right]}$ reflects the fact that in PCO the mobility of localized electrons sitting on Pr^3+^ sites is only possible if adjacent available Pr sites are “empty” (Pr^4+^ state). The measured *E_a_* in the space charge regions is thus expected to have multiple contributions, which include the sum of one-third of the reduction enthalpy of Pr in PCO, the small polaron hopping energy, and a contribution from the space charge potential. Directly solving these equations can become challenging especially in the scenario that $\Delta\phi>0$ where we can not simplify the site occupancy term $\left( 1-\frac{\left[ {Pr}^{3+} \right]\left( x \right)}{\left[ {Pr}_{tot} \right]} \right)$. Moreover, one of the major assumptions in deriving the Gouy-Chapman model no longer holds, i.e., the assumption of a dilute limit condition. Nevertheless, for the sake of our study, we can visually inspect equations (3) and (4) to predict the expected behavior.

When $\Delta\phi<0$, a situation described at the substrate/surface heterointerface of our nanowire in contact with an acidic oxide, then $\frac{\left[ {Pr}_{Ce}^{'} \right]\left( x \right)}{\left[ {Pr}_{Ce}^{'} \right]_{bulk}}<1$ and $\left[ {Pr}_{Ce}^{'} \right]\left( x \right)$ becomes depleted in the vicinity of the interface. Conduction along $\sigma_{//,sc}$ would systematically be bypassed by the parallel contribution of $\sigma_{bulk}$ (**Figure S7a**).

When $\Delta\phi>0$, a situation described at the substrate/surface heterointerface when our nanowire is in contact with a basic oxide or at the grain boundaries, then an accumulation of ${Pr}^{3+}$ in the space charge region is expected to occur (**Figure S7b**). In the accumulated space charge region, where $\left[ {Pr}^{3+} \right]\left( x \right) \sim50\%\left[ {Pr}_{tot} \right],$then a local maximum in conductivity is expected, where $\left[ {Pr}^{3+} \right]\left( x \right)\left( 1-\frac{\left[ {Pr}^{3+} \right]\left( x \right)}{\left[ {Pr}_{tot} \right]} \right)$, is maximized. Above this point, closer to the interface, the effective mobility of small polaron is expected to decrease with further increases in $\left[ {Pr}^{3+} \right]\left( x \right)$, owing to the decrease in site availability (i.e. $\left[ {Pr}^{3+} \right]\left( x \right)\left( 1-\frac{\left[ {Pr}^{3+} \right]\left( x \right)}{\left[ {Pr}_{tot} \right]} \right)$ . The implication is that an anisotropic conductivity profile may arise, with a high resistivity region developing close to the interface wherever $\left[ {Pr}^{3+} \right]\left( x \right)>50\%[{Pr}_{tot}]$, contributing to a series resistance for perpendicular transport across the interface, while a conductivity maxima where $\left[ {Pr}^{3+} \right]\left( x \right) \sim50\%[{Pr}_{tot}]$, occurring a small distance away from the interface for parallel transport. The conductivity profile will follow the trends displayed in **Figure S8**:

The above rationale explains how electron accumulation at a heterointerface can enable enhanced conductivity parallel to the interface (for MgO and Li_2_O), while causing higher resistance for bulk transport across grain boundaries. It is also entirely consistent with the observations made previously by Zhang et al. in bulk nanocrystalline ceramic of PCO, when we consider that ceramic samples sintered at higher temperatures typically exhibit smaller grain boundary space charge potentials.^[10]^ Nanocrystalline samples sintered at lower temepratures are therefore expected to have higher space charge potentials and therefore stronger Pr^3+^ accumulation creating a more significant resistive blockage to perpendicular transport across the space charge region, even though the apparent activation energy is reduced.

Moreover, we recently showed that the GB space charge potentials in acceptor-doped CeO_2_ thin films could be modulated through the in-diffusion of elements from the substrates at intermediate temperature (700-900 °C).^[13]^ We showed that depending on the element diffusing up to the GB (Al/Mg), increases or decreases in space charge potentials would be observed. This was enabled by the fact that cation diffusion along GB cores is accelerated relative to the bulk at these intermediate temperatures and the fact that Al^3+^ ions are believed to sit on interstitial sites in GBs in ceria-based ceramics, inducing a net positive charge of 3+ ${(Al}_{i}^{\ldots})$ and therefore an increase in space charge potential,^[14–16]^ while Mg^2+^ sits substitutionally, leading to a net negative charge of 2- (${Mg}_{Ce}^{''})$.^[13]^ The up-diffusion of Mg^2+^ would be expected to decrease the space charge potential at the grain boundaries, while leading to a depletion of electrons at the substrate/surface interface. This grain boundary in-diffusion process would also explain why transport across grain boundaries will be more resistive than the bulk while possessing a lower activation energy, as the sign of the space charge potential would actually act to reduce the total activation of conductivity composed of one-third of the reduction enthalpy and bulk mobility (note that in equation(1) and equation (2), the exponential terms have different signs). In the case of **Figure S9**, where the PCO_NA_ on Al_2_O_3_ is annealed at 700 °C, Al^3+^ at the grain boundary is expected to cause a highly increased space charge potential and therefore a higher series resistance ($\rho_{\perp}$) induced by the depressed small polaron mobility, contributing to a lower conductance. On the other hand at 400 °C, the space charge potential at the grain boundaries is expected to be small and therefore the bulk conductivity of the film would align closer with bulk theoretical expectations. A consistent explanation can also be applied to the case in which MgO is used as the substrate (**Figure S9**). When PCO_NA_ on MgO is annealed at 700 °C, Mg^2+^ at the grain boundary is expected to cause a reduced space charge potential, thereby facilitating conduction perpendicular to the grain boundaries and contributing to a higher conductance. In the case of the sample annealed at 400 °C, the space charge potential at the grain boundaries is expected to remain relatively large (intrinsic bulk), resulting in lower conductivity. In **Figure S8**, we provide a 3D visualization of the anisotropic charge transport that would occur at surface and grain boundaries due to a $\Delta\phi>0$ and compare it to the situation of electron depletion at the substrate interface (i.e. $\Delta\phi<0$).

**Note S3. Influence of insulating substrate acidity on 3 mol% Gd-doped ceria (GDC3) nanowire arrays ionic properties.**

In the case of GDC3, where ionic conduction is the dominant transport mechanism, which occurs through oxygen vacancy hopping, the conduction behavior differs from that of PCO_NA_. Specifically, when a relatively basic MgO was used as a substrate, oxygen vacancy depletion was induced, leading to a total decrease in conductivity. On the other hand, the Al_2_O_3_ (relatively acidic) substrate promoted vacancy accumulation, resulting in a total increase in conductivity. In contrast to PCO, where bulk transport is composed of contributions from thermal carrier generation and their migration, in GDC3, the bulk conductivity is composed only of the migration energy of the oxygen vacancies (- 0.7 eV) as the carrier concentration is fixed by the aliovalent Gd^3+^ dopant, while for polycrystalline samples, activation energies > 1 eV are typically reported, associated with the additional grain boundary series resistance caused by the space charge barriers with positive potentials barriers.^[11,13]^

The ionic properties of GDC3 nanowire arrays on two different substrates were characterized through Arrhenius plots in **Figure S11**. In this case, both samples were annealed at 400 °C, so the GB space charge potential driven by cation in-diffusion from substrate can be ignored. GDC3 nanowire arrays on the MgO substrate exhibited similar *E_a_* (1.23 eV) comparable to that of polycrystalline GDC3 thin film. However, GDC3 nanowire arrays on the Al_2_O_3_ substrate exhibited a lower *E_a_* of 1.01 eV. Moreover, GDC3 nanowire arrays on the Al_2_O_3_ showed higher in-plane conductivity than those on the MgO substrate.

These results are attributed to the conduction pathways determined by the acidity-mediated space charge potential at the heterointerface. In the case of MgO, a resistive heterointerface is formed, causing conduction to occur through the bulk (R_1_). On the other hand, in the case of Al_2_O_3_, oxygen vacancy accumulation facilitates conduction through the substrate heterointerface (R_2_), which is opposite to the tendency observed in PCO_NA_. Furthermore, for Al_2_O_3_, the electric fields generated by the space charge at the heterointerface (due to a negative space charge potential) are opposite directions to the positively charged space charge at the GB, partially compensating each other and leading to a reduced *E_a_* at the grain boundary heterointerface triple junction.

**Note S4. Comparison of polycrystalline and epitaxial PCO20 films to elucidate grain boundary in-diffusion effects**

Polycrystalline and epitaxial PCO20 films were fabricated using PLD with a PCO20 target on Al_2_O_3_ substrate by modulating the temperature and deposition rate. For the polycrystalline PCO20 film, deposition was conducted at room temperature with a laser frequency of 10 Hz. In contrast, the epitaxial film was deposited at 700 °C with a reduced frequency of 2 Hz, while all other deposition conditions were maintained constant.

Evidence for the role of GB space charge potentials in impacting the effective conductivity of polycrystalline films can be obtained by comparing the AC impedance analysis of polycrystalline and epitaxial PCO20 films fabricated by PLD. We note that similarly grown polycrystalline and epitaxial films of PCO20 exhibited a trend (decrease in conductivity and activation energy) when moving from the epitaxial film to polycrystalline film, though the activation energies were generally lower than for the PCO20 bulk reference, likely due to lattice strain. In polycrystalline films, numerous GBs are present, allowing Al to diffuse into them during annealing. In contrast, in epitaxial films without GBs, such space charge modulation is prevented even under high-temperature annealing conditions (**Figure S13d**). As verified by XRD analysis (**Figure S13a and 13b**), the epitaxial films predominantly exhibit (111) and its family of planes, while polycrystalline films display a mix of planes, including (111) and (200). In addition, the shifted positions of peaks appearing in both polycrystalline and epitaxial films compared to PCO_NA_ imply tensile strain, which is reported to decrease the migration energy and improve diffusivity of oxygen vacancy hopping.^[10,17]^ As expected, the total conductivity of the epitaxial film is higher than that of the polycrystalline film due to increased blocking at the polycrystalline film’s GBs. Furthermore, the Arrhenius plots reveal that the *E_a_* of the polycrystalline film is lower than that of epitaxial films associated with the space charge regions surrounding the GBs, consistent with the PCO_NA_ results (**Figure S13c**).

**Note S5.** Reported studies where substrate acidity effects could potentially influence as a contributing factor.

[1] J. Garcia-Barriocanal, A. Rivera-Calzada, M. Varela, Z. Sefrioui, E. Iborra, C. Leon, S. J. Pennycook, J. Santamaria, *Science* 2008, **321**, 676.

[2] L. Yan, P. A. Salvador, *ACS Appl. Mater. Interfaces* 2012, **4**, 2541.

[3] Z. Othmen, A. Schulman, K. Daoudi, M. Boudard, C. Acha, H. Roussel, M. Oueslati, T. Tsuchiya, *Appl. Surf. Sci.* 2014, **306**, 60.

[4] B. W. Veal, S. K. Kim, P. Zapol, H. Iddir, P. M. Baldo, J. A. Eastman, *Nat. Commun.* 2016, **7**, 11892.

[5] G. Hamaoui, N. Horny, C. L. Gomez-Heredia, J. A. Ramirez-Rincon, J. Ordonez-Miranda, C. Champeaux, F. Dumas-Bouchiat, J. J. Alvarado-Gil, Y. Ezzahri, K. Joulain, M. Chirtoc, *Sci. Rep.* 2019, **9**, 8728.

[6] A. Atul, M. Ahmadi, P. Koutsogiannis, H. Zhang, B. J. Kooi, *Adv. Mater. Interfaces* 2024, **11**, 2300639.

**Table S1.** Measurement of Pr chemical composition of PCO_NA_ fabricated using a PCO 20 at % target, through inductively coupled plasma mass spectrometer (ICP-MS). The reported loading amount of Pr in the PCO_NA_ represents the mean ± standard deviation (*n* = 3)

| **Sample** | **ICP-MS (at %)** |
| --- | --- |
| PCO_NA_ (Fabricated using PCO 20 at % target) | 19.15 $\pm$ 0.1 |

**Table S2.** Absolute conductivity values for the binary oxides (Li_2_O and SiO_2_) at 600 °C used in this work.

|  | **Conductivity (S/cm) at 600 °C** |
| --- | --- |
| Li_2_O (for basic)^[18]^ | - 10^-5^ |
| SiO_2_ (For acidic)^[19]^ | - 10^-12^ |

**
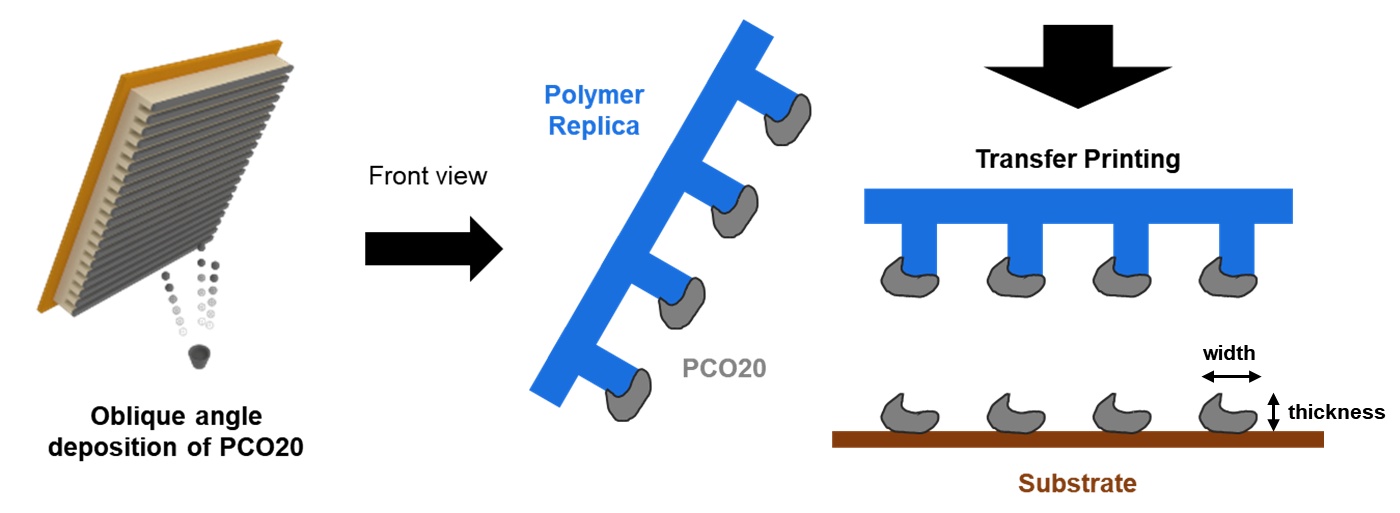
**

**Figure S1.** Schematic illustration of nanowire cross-sections formed through solvent-assisted nanotransfer printing (during oblique angle deposition), which result in the “drop-with-tail” features. In this study, the employed PCO nanowires have a width of 50 nm and a thickness of 50 nm.

**
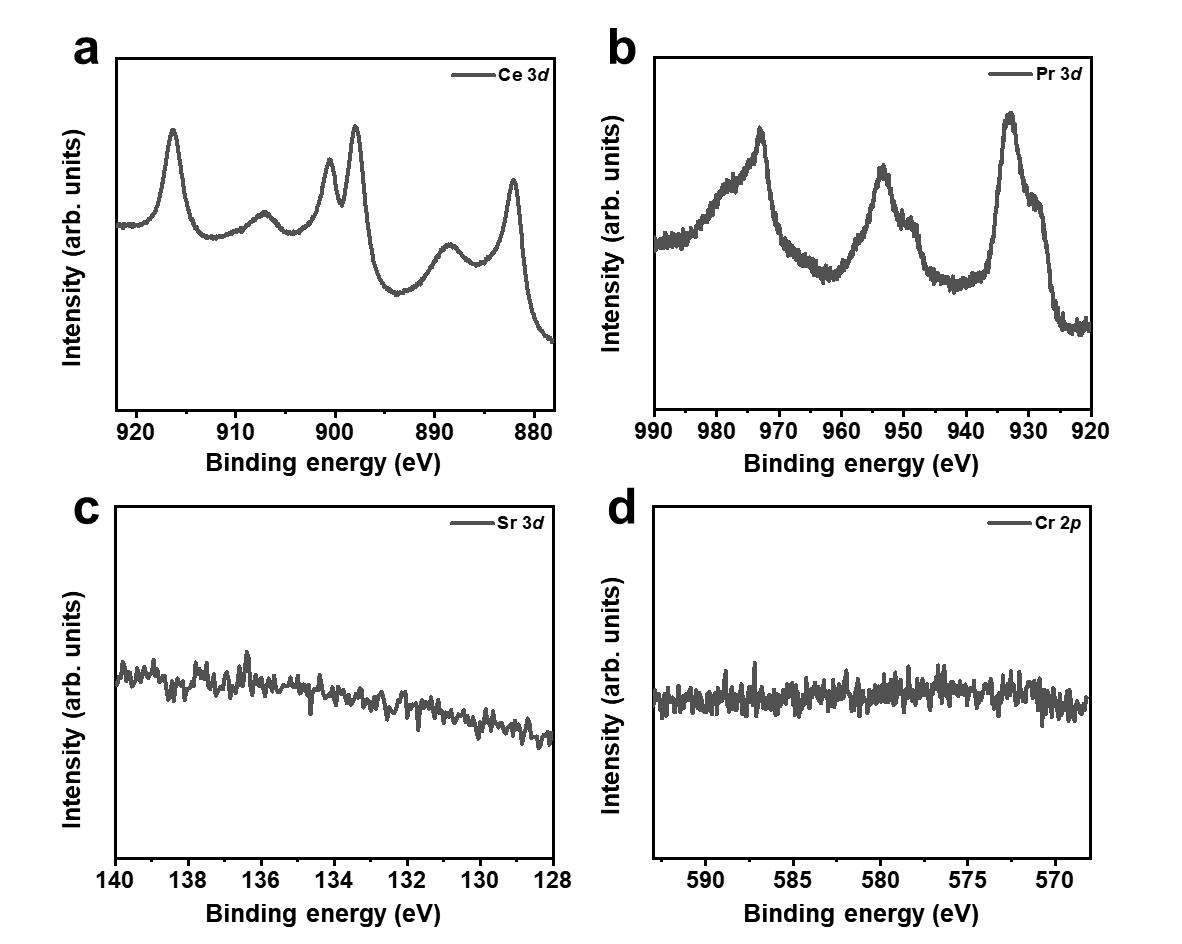
Figure S2.** X-ray photoelectron spectroscopy (XPS) spectra of pristine PCO_NA_ with the different binding energy regions of (a) Ce 3*d*, (b) Pr 3*d*, (c) Sr 3*d*, and (d) Cr 2*p* spectra, respectively. XPS data exhibits no presence of Sr or Cr elements at the surface which could cause surface degradation in PCO_NA_ sample.


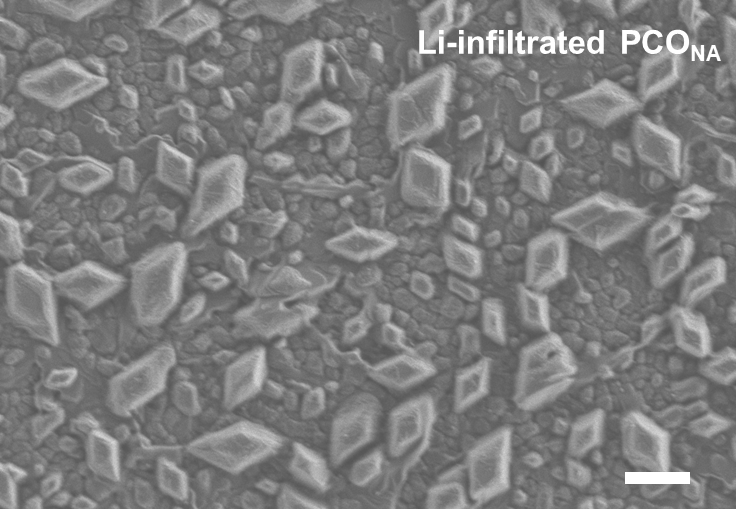


**Figure S3.** SEM image of Li-infiltrated PCO_NA_ fabricated on Al_2_O_3_. The PCO_NA_ is observed to be fully covered by Li_2_O given infiltration with a concentrated solution. (scale bar, 2 $\mu$m).

**
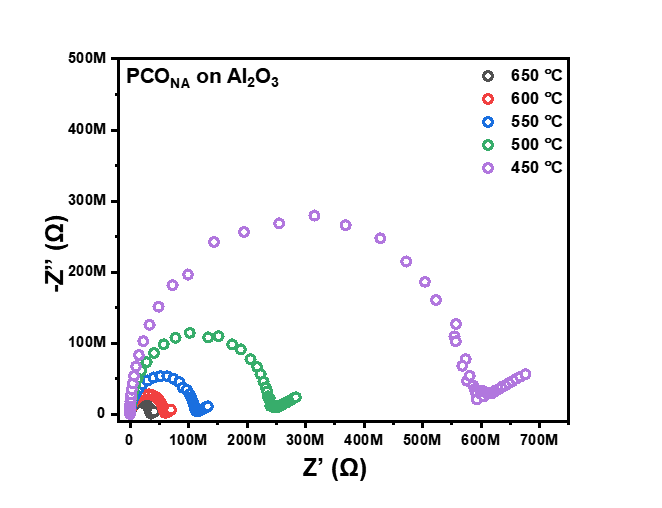
Figure S4.** Impedance spectra measured from 450 °C to 650 °C on uninfiltrated PCO_NA_ fabricated on Al_2_O_3_ insulating substrate.

**
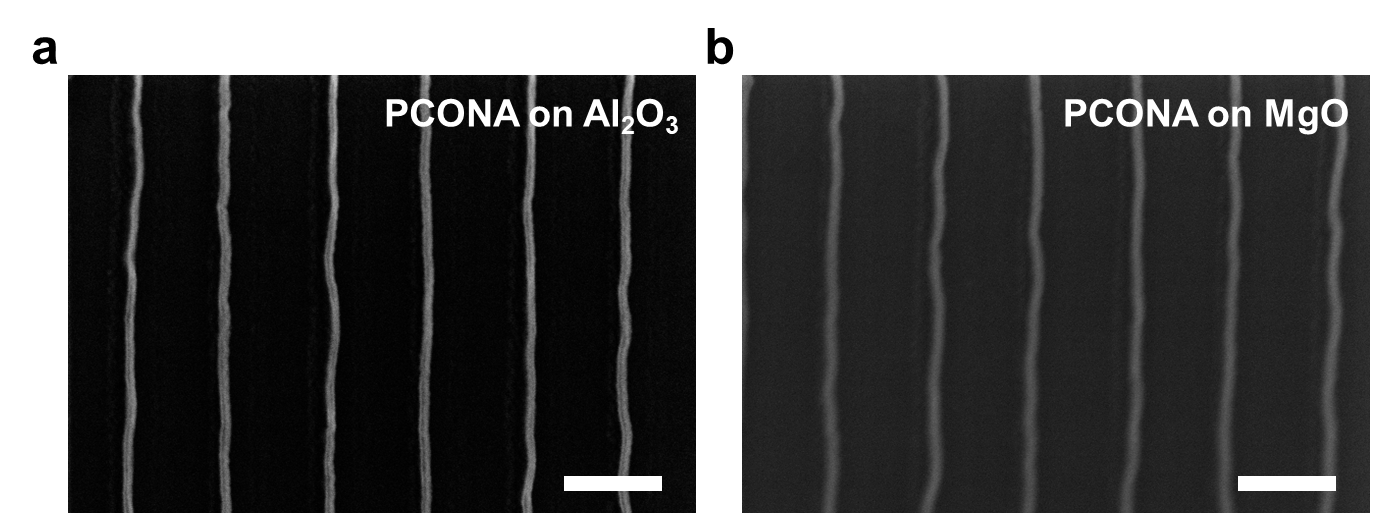
Figure S5.** Scanning electron microscopy (SEM) images of PCO_NA_ fabricated on (a) Al_2_O_3_ and (b) MgO substrates, respectively (scale bar, 200 nm).


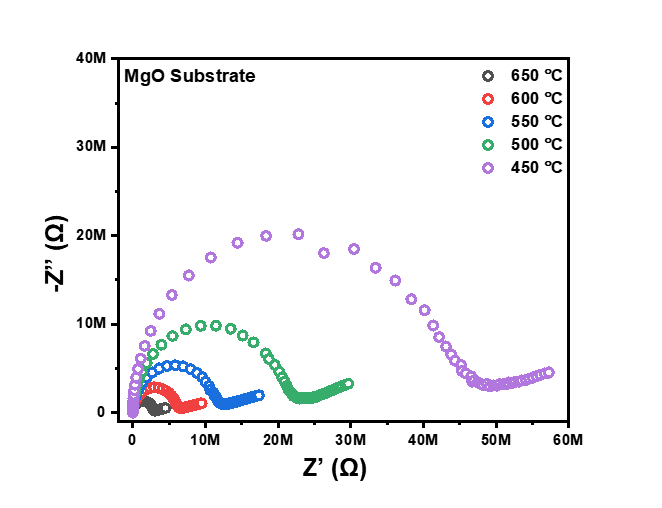
**Figure S6.** Impedance spectra measured from 450 °C to 650 °C on uninfiltrated PCO_NA_ fabricated on MgO insulating substrate.


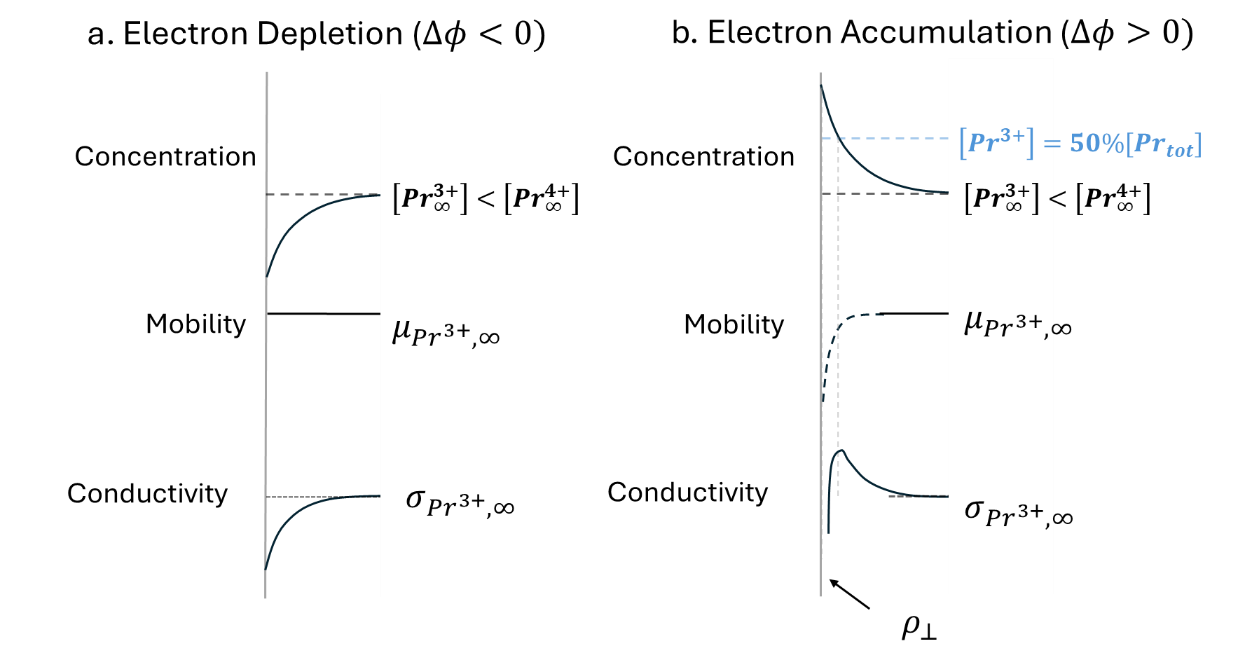
**Figure S7.** Illustration of space charge model for the case of (a) positive and (b) negative space charge potential and its impact on the spatial distribution in space charge region of ${Pr}^{3+}$concentration ($\left[ {Pr}^{3+} \right]$), effective mobility $\mu_{Pr,effective} \sim\left[ {Pr}^{3+} \right]\left( x \right)\left( 1-\left[ {Pr}^{3+} \right]\left( x \right) \right)\mu_{Pr}$ and expected conductivity ($\sigma$).


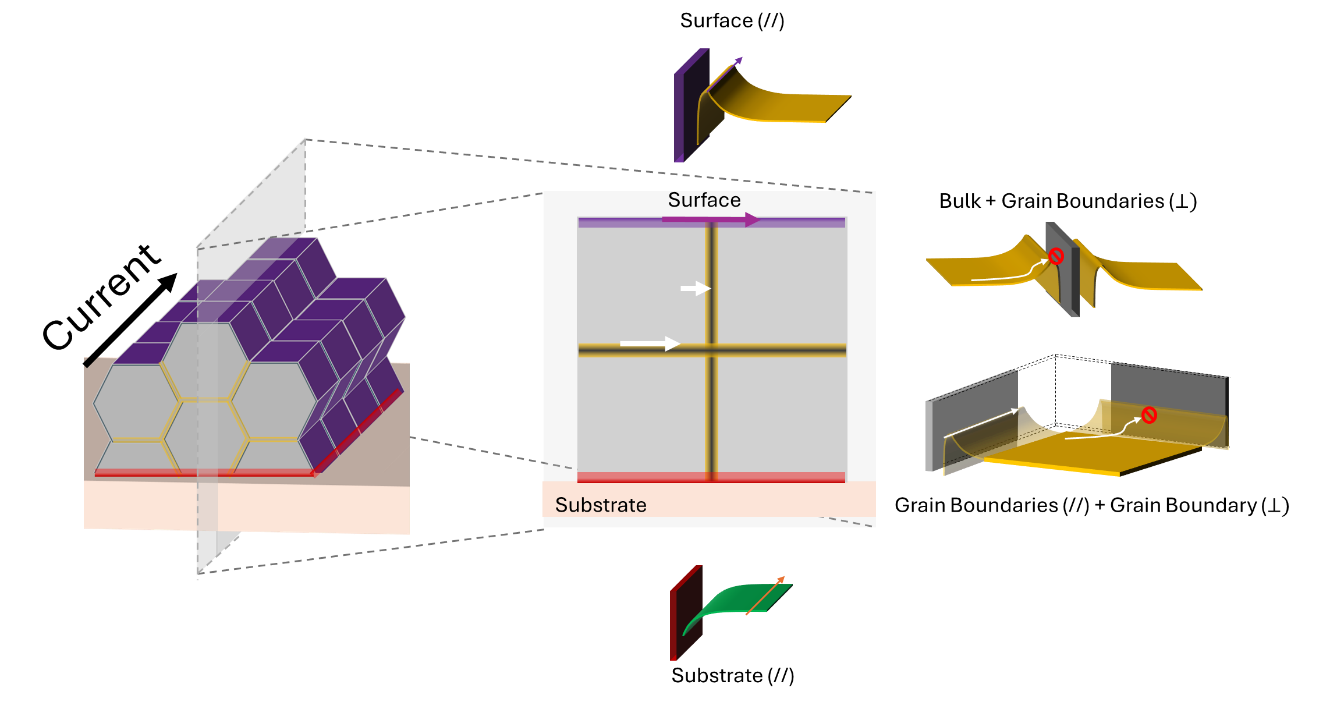


**Figure S8.** 3D visualization of nanowire composed of multiple nanoscale grains. (left image) cross section of a nanowire – (center image) along nanowire. Purple shaded and gray areas surrounded by yellow shaded areas, represent the surface in contact with a base and grain boundaries where $\Delta\phi>0$, causing an anisotropic profile of $\left[ {Pr}^{3+} \right]\left( x \right)$ enabling parallel conduction to the surface ($//)$, while causing resistive conduction across the grain boundaries ($\perp$). Red shaded areas at the bottom in contact with substrate represent case of nanowire in contact with acidic susbtrate (i.e. Al_2_O_3_), expected to cause $\Delta\phi<0$ and therefore electron depletion.

**
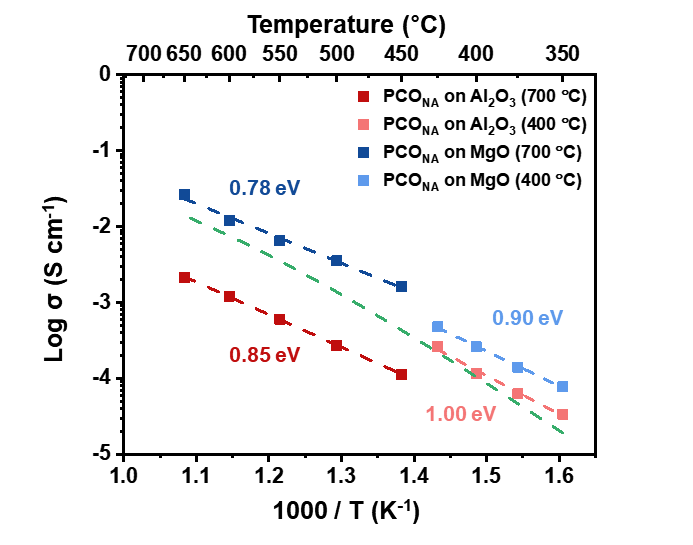
Figure S9.** Arrhenius plots of in-plane conductivity of PCO_NA_ fabricated on Al_2_O_3_ and MgO substrates annealed at 400 °C and 700 °C, respectively. The green dashed line indicates the conductivity behavior of bulk PCO20 calculated based on previously reported defect chemical and kinetic modelling.^[20]^

**Figure S10.** XRD patterns of PCO_NA_ fabricated on Al_2_O_3_ substrate annealed at 400 °C and 700 °C, respectively.**
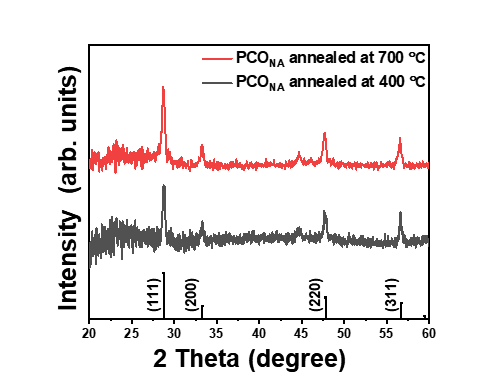
** Black vertical lines at bottom of figure indicate the dominant facets of the cerium oxide crystal structure.

**
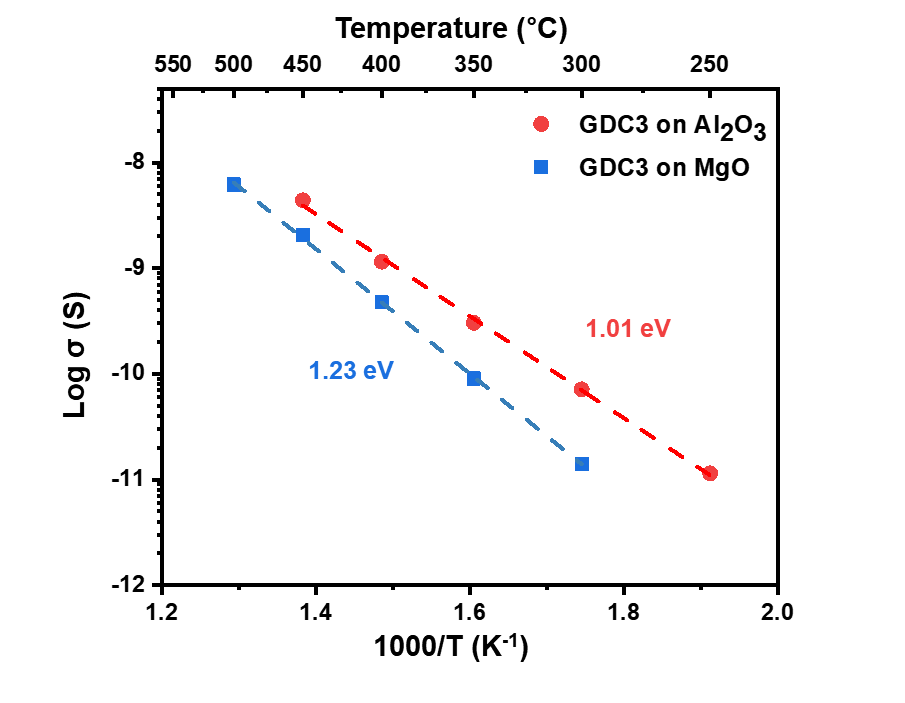
Figure S11.** Arrhenius plots of in-plane conductivity of 3 at% Gd-doped ceria (GDC3) nanowire arrays fabricated on Al_2_O_3_ and MgO substrates annealed at 400 °C along with their respective activation energies.

**
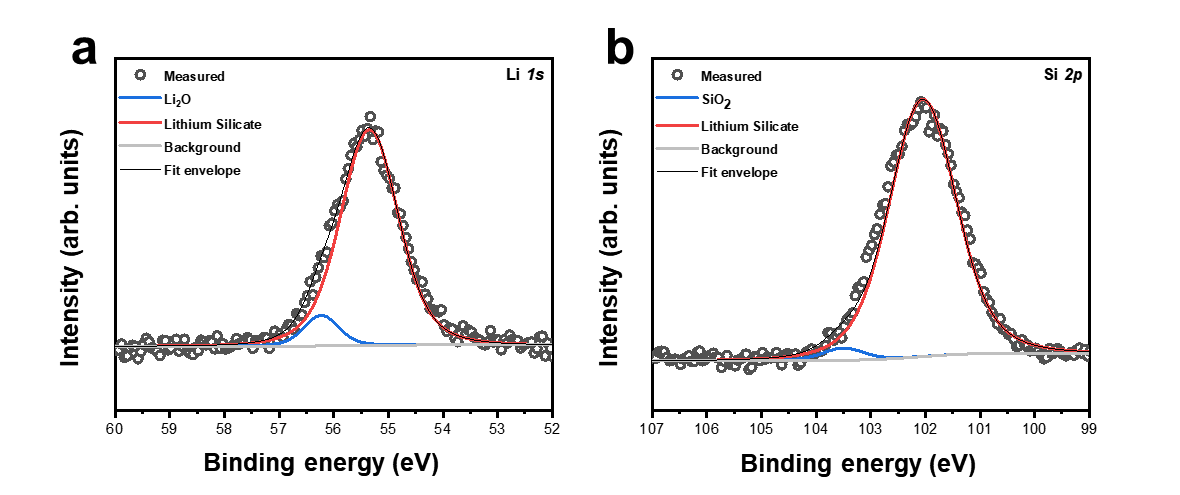
Figure S12.** XPS spectra of PCO_NA_ after sequential infiltration with Li-species following Si-species, showing the different binding energy regions of (a) Li *1s* and (b) Si *2p* spectra, respectively.

**
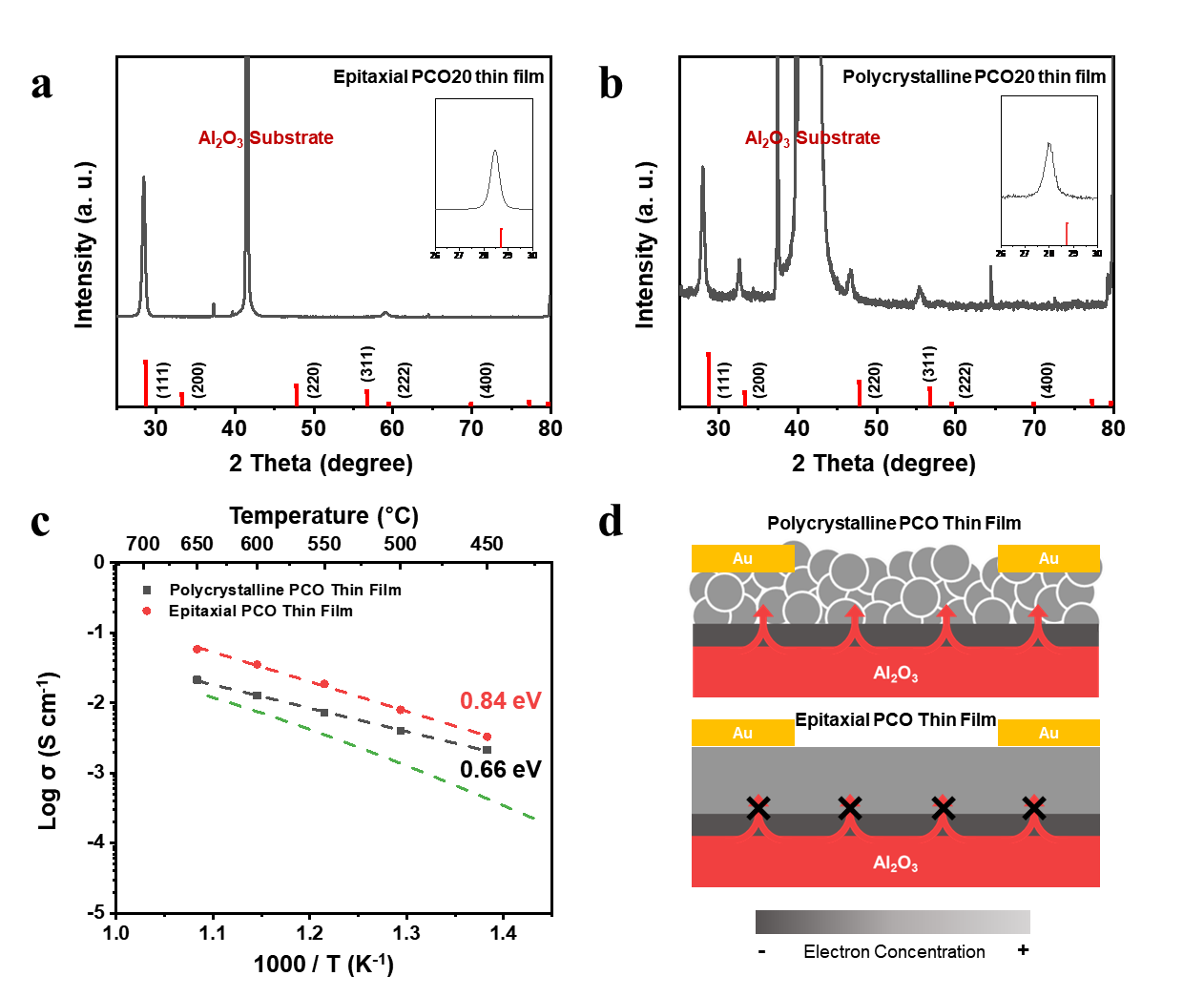
****Figure S13.** XRD patterns of (a) epitaxial and (b) polycrystalline PCO20 thin films deposited on Al_2_O_3_ insulating substrates via pulsed laser deposition (PLD). The inset images show enlarged views of the spectra from 26° to 30°, reavealing a peak shift due to tensile strain. Red vertical lines at bottom of figures indicate dominant facets of cerium oxide crystal structure. (c) Arrhenius plots of in-plane conductivity of epitaxial and polycrystalline PCO20 thin films, along with their respective activation energies. The green dashed line indicates the conductivity behavior of a bulk PCO20 film calculated based on previously reported defect chemical and kinetic modelling.^[20]^ (d) Comparison between polycrystalline and epitaxial PCO20 thin films. Substrate heterointerface shown in black corresponds to electron depletion due to relative acidity of Al_2_O_3_. Diffusion of Al ions only occurs in the polycrystalline PCO20 thin film, leading to GB electron accumulation, depicted in white.

References

[1] R. Takada, H. Yao, *J. Phys. Chem. C* 2025, ***129***, 5461.

[2] B. Koo, K. Kim, J. K. Kim, H. Kwon, J. W. Han, W. C. Jung, *Joule* 2018, ***2***, 1476.

[3] H. Kim, H. G. Seo, S. Ahn, H. L. Tuller, W. C. Jung, *J. Mater. Chem. A* 2025, ***13***, 9708

[4] W. Jung, H. L. Tuller, *Energy Environ. Sci.* 2012, ***5***, 5370.

[5] H. G. Seo, D. H. Kim, J. Seo, S. J. Jeong, J. Kim, H. L. Tuller, J. W. Son, W. C. Jung, *Adv. Energy Mater.* 2022, ***12***, 2202101.

[6] S. R. Bishop, T. S. Stefanik, H. L. Tuller, *J. Mater. Res.* 2012, ***27***, 2009.

[7] H. J. Han, G. R. Lee, Y. Xie, H. Jang, D. J. Hynek, E. N. Cho, Y. J. Kim, Y. S. Jung, J. J. Cha, *Sci. Adv.* 2021, ***7***, eabh2012.

[8] N. Shibata, A. Goto, S. Y. Choi, T. Mizoguchi, S. D. Findlay, T. Yamamoto, Y. Ikuhara, *Science* 2008, ***322***, 570.

[9] J. Schou, *Appl. Surf. Sci.* 2009, ***255***, 5191.

[10] S. Zhang, Z. Fang, M. Chi, N. H. Perry, *ACS Appl. Mater. Interfaces* 2024, ***17***, 898.

[11] X. Guo, R. Waser, *Prog. Mater. Sci.* 2006, ***51***, 151.

[12] X. Tong, D. S. Mebane, R. A. De Souza, *J. Am. Ceram. Soc.* 2020, ***103***, 5.

[13] T. Defferriere, Y. B. Kim, C. Gilgenbach, J. M. LeBeau, W. Jung, H. L. Tuller, 2025, *arXiv:*2504.10684

[14] X. Xu, C. Carr, X. Chen, B. D. Myers, R. Huang, W. Yuan, S. Choi, D. Yi, C. Phatak, S. M. Haile, *Adv. Energy Mater.* 2021, ***11***, 2003309.

[15] L. Minervini, M. O. Zacate, R. W. Grimes, *Solid State Ion.* 1999, ***116***, 339.

[16] X. Xu, Y. Liu, J. Wang, D. Isheim, V. P. Dravid, C. Phatak, S. M. Haile, *Nat. Mater.* 2020, ***19***, 887.

[17] G. F. Harrington, D. Kalaev, B. Yildiz, K. Sasaki, N. H. Perry, H. L. Tuller, *ACS Appl. Mater. Interfaces* 2019, ***11***, 34841.

[18] S. Lorger, R. Usiskin, J. Maier, *J. Electrochem. Soc.* 2019, ***166***, A2215.

[19] J. K. Srivastava, M. Prasad, J. B. Wagner, *J. Electrochem. Soc.* 1985, ***132***, 955.

[20] S. R. Bishop, T. S. Stefanik, H. L. Tuller, *J. Mater. Res.* 2012, **27**, 2009.
